# An investigation of the effect of *Zataria multiflora* Boiss and *Mentha piperita* essential oils to improve the chemical stability of minced meat

**DOI:** 10.14202/vetworld.2018.1656-1662

**Published:** 2018-12-11

**Authors:** Mojtaba Raeisi, Mohammad Hashemi, Majid Aminzare, Asma Afshari, Tayebeh Zeinali, Behrooz Jannat

**Affiliations:** 1 Infectious Disease Research Center, Golestan University of Medical Sciences, Gorgan, Iran; 2 Department of Nutrition, Faculty of Health, Golestan University of Medical Sciences, Gorgan, Iran; 3 Department of Nutrition, Faculty of Medicine, Mashhad University of Medical Sciences, Mashhad, Iran; 4 Department of Food Safety and Hygiene, Faculty of Public Health, Zanjan University of Medical Sciences, Zanjan, Iran; 5 Social Determinants of Health Research Center, School of Health, Birjand University of Medical Sciences, Birjand, Iran; 6 Halal Research Center of IRI, FDA, Tehran, Iran

**Keywords:** antioxidant, *Mentha piperita*, minced meat, *Zataria multiflora* Boiss

## Abstract

**Background and Aim:**

Extending the shelf life of foods is an essential concept in food safety. Most of the time, foods deteriorate through the growth of microorganisms or oxidation process. Essential oils (EOs) derived from plant material have well-documented antioxidant and antibacterial activity. This study aimed to evaluate the effect of *Zataria multiflora* Boiss EO (ZEO) and *Mentha piperita* EO (MEO) on the chemical stability of minced meat during storage at 7°C.

**Materials and Methods:**

Total phenolic content, β-Carotene bleaching test, ferric reducing antioxidant potential assay, and 2,2-Diphenyl-1-picrylhydrazyl radical scavenging activity were used to determine the antioxidant potential of EOs. Five different groups including control, ZEO 0.3%, ZEO 0.5%, MEO 0.3%, and MEO 0.5% were designed to assess the chemical stability of minced meat by measuring pH, thiobarbituric acid (TBA), total volatile base nitrogen (TVBN), and peroxide value (PV).

**Results:**

pH did not have any significant change during storage. TBA values in the control group were significantly higher than the treatment groups, especially from the 5^th^ day of storage. TVBN in the treatment group was significantly lower than the control group during storage. PV values in the treatment group were significantly lower than the control group during storage.

**Conclusion:**

Results indicate that ZEO and MEO had an excellent antioxidant activity and retarded the spoilage process in minced meat.

## Introduction

Food spoilage which is mainly caused by the oxidation process and growth of microorganisms during storage and marketing is one of the most important aspects of food safety and extending the shelf life of food [1,2]. Peroxidation of lipids leads to the formation of off-flavors and decrease the quality of the foods. In the food industry to extend the shelf life of food, some chemical additives such as butylated hydroxyanisole (BHA), butylated hydroxytoluene (BHT), and propyl gallate are usually mixed with foods having a high-fat content [3]. Therefore, due to growing concerns of consumers about the teratogenic or carcinogenic effects of chemical additives in foods, use of natural compounds such as essential oils (EOs) bear in mind [1,4,5].

EOs are the secondary metabolites of aromatic plants with a strong odor and complex structure. They are considered natural sources of antimicrobial and antioxidant compounds [6]. Food and Drug Administration approved EOs as generally recognized as safe [7]. Many studies showed the antimicrobial and antioxidant activity of EOs [3,8-10]. Antioxidants can act as a scavenger of free radicals and inhibit the oxidation process. Consumption of natural antioxidants decreased the risk of cancer, cardiovascular disease, diabetes and diseases associated with aging [11]. Therefore, searching for natural, effective and safe antioxidants which can retard the progress of many chronic diseases and protect the human body by neutralizing free radicals is increased [11]. Plants containing EOs are a potential source of food additives in the food industry.

*Mentha piperita* (peppermint) is one of the members of the *Mentha* species. This plant is widely grown in temperate areas, but nowadays, it is cultivated for the production of EO [12]. In recent years, peppermint was used in different kinds of matrices such as perfume, cosmetic, pharmaceutical, and food industries. Many studies have proved the antibacterial and antioxidant activity of peppermint [3,13,14]. *Zataria multiflora* Boiss is widely used as a flavoring agent in several foods [9,15,16]. Two main components of *Z. multiflora* are thymol and carvacrol that had been reported to have antibacterial, antioxidant, antiseptic, and antifungal properties [4,6,8,10].

Minced meat is a highly oxidizable food. Addition of synthetic antioxidants raises some concerns about its safety. Seeking new ways to retard the oxidation process and spoilage is of most importance. According to the best of our knowledge, it is the first study to evaluate the chemical stability of minced meat with the *Z. multiflora* and *M. piperita* at abuse refrigerated temperature. Thus, the present study aimed to evaluate and compare the antioxidant activity of *M. piperita* EOs (MEOs) and *Z. multiflora* EOs (ZEOs) and chemical quality of minced meat during 9 days storage at 7°C.

## Materials and Methods

### Ethical approval

This study was not involved any human or animal subject and no ethical approval was required.

### EOs preparation and analysis

The plants of *Z. multiflora* and *M. piperita* were purchased from local groceries of Gorgan, Iran and authenticated at Gorgan University of Agricultural Sciences and Natural Resources. Using a Clevenger-type apparatus, dried aerial parts of the plants were hydrodistilled for 3 h and the oil was dehydrated with sodium sulfate and stored in the dark at 4°C for further analysis according to the method previously described [14,17]. The chemical composition of ZEOs and MEOs was analyzed by a gas chromatograph (Hewlett-Packard, Santa Clara, CA; 6890N) including a column HP-5MS (30 m length×0.25 mm i.d., film thickness 0.25 mm) and equipped with a mass spectrometer (Hewlett-Packard 5973N). The gas chromatograph program was as follows: Helium flow rate was 1.5 mL/min, and temperature increased from 40°C to 240°C with a gradient of 3°C/min. The initial and final temperature was held for 6 min (min) followed by an increase to 300°C for 15°C/min holding for 3 min. Injector port and detector temperatures were 290°C and 250°C, respectively. Identification of the spectra was carried out using the Willey-229 mass database, retention time, calculating the Kovats’ index, the mass spectrum analysis of compounds, and comparison with standard mass spectra and valid sources such as National Institute of Standards and Technology [8,9].

### Total phenolic content

Total phenolic content of the EOs was determined using the method previously described by former studies with some modifications [8,18]. Briefly, EOs (0.5 ml) and Folin–Ciocalteu’s reagent (0.5 ml) were mixed. An amount of 2.5 ml of sodium carbonate solution (1N) was added to the mixture after 3 min and adjusted to 10 ml with distilled water, and the absorbance was read at 725 nm after an incubation period of 2 h. The content of phenol was calculated as a gallic acid equivalent (GAE) from the calibration curve of gallic acid standard solutions (0–0.1 mg/ml) and expressed as mg GAEs per g of dried plant.

### β-Carotene bleaching (BCB) test

An amount of 0.1 mg β-Carotene, 20 mg linoleic acid and 100 mg Tween 40, all dissolved in chloroform were added to a flask. The chloroform was then evaporated, under vacuum at 50°C by a rotary evaporator, and 50 mL oxygenated distilled water was added to the mixture, and emulsion A was formed after 1 min emulsification in a bath sonicator. Then, 200 μL of each EO was mixed with 5 mL of emulsion A, in open-capped cuvettes. A control was prepared without antioxidant, consisting of 200 μL of ethanol and 5 mL of emulsion A. A second emulsion (B) consisting of 20 mg linoleic acid, 100 mg Tween 40 and 50 mL oxygenated water was also prepared. Ethanol (200 μL) was added to 5 mL of emulsion B and used to zero the spectrophotometer. The absorbance of the samples was read immediately (t=0) and every 15 min intervals for 120 min on a CECIL9000 spectrophotometer at 470 nm. The cuvettes were thermostated at 50°C between measurements. The average percent of inhibition was calculated by the following formula [19,20].

I %=(A β-carotene after 2h assay/A Initial β-carotene)×100

### Ferric reducing antioxidant potential (FRAP) assay

About 10 mL of acetate buffer (300 mM, pH 3.6) (3.1 g sodium acetate trihydrate) mixed with 1.0 mL of ferric chloride hexahydrate 20 mM (dissolved in distilled water) and 1.0 mL of 2,4,6-tri-(2-pyri-dyl)-s-triazine 10 mM (dissolved in HCl 40 mM) to prepare the FRAP reagent. Five different concentrations: 100, 50, 25, 12.5, and 6.25 mg/mL of EO (10 μL) mixed to 190 μL of the FRAP solution in a 96-well plate, and incubated at 37°C for 30 min. The absorbance of the reaction mixture read at 593 nm by use of a BioTek microplate reader (Synergy H4, USA). All experiments were repeated 3 times. A standard curve was produced by the use of gallic acid as reference material. Data were expressed as milligram GAE per gram dry weight (DW) by the dried sample (mg GAE/g DW basis) [20,21].

### 2,2-Diphenyl-1-picrylhydrazyl (DPPH) radical scavenging activity

DPPH, a stable free radical was used to determine the radical scavenging ability of the EO. EO solutions (at five levels: 100, 50, 25, 12.5, and 6.25 mg/mL) added to freshly prepared DPPH solution. After severe shaking, the mixture was left at room temperature for 60 min. The decrease of the absorbance at 517 nm showed the reduction of DPPH radical. The degree of DPPH discoloration was showed the efficiency of DPPH radical scavenging and calculated using the following equation: Percentage scavenging effect/% Inhibition=[(A_DPPH_–A_S_)/A_DPPH_)×100].

Where A_S_ is the absorbance of the solution containing the sample extract with a particular level; A_DPPH_: The absorbance of the DPPH solution. The extract concentration providing 50% inhibition (IC_50_) was calculated from the graph of the scavenging effect percentage against extract concentration in the solution [22].

### Preparation of minced meat containing ZEO and MEO

The beef meat was minced in a meat grinder and divided into five groups according to the following treatments: Control (no addition), 0.3% v/w MEO, 0.5% v/w MEO, 0.3% v/w ZEO, and 0.5% v/w ZEO. After appropriate homogenization, the samples were separately packed in UV-sterilized polyethylene bags and stored at 7°C for subsequent analysis on days: 0, 3, 5, 7, and 9. Distilled water was added in control samples instead of EO.

### pH measuring

pH values were measured by use of a pH meter (pH 510 Eutech; CyberScan, Ayer Rajah, Singapore) [15].

### Peroxide value (PV)

PV analysis was performed according to Ehsani *et al*. [15]. Results were expressed as milliequivalents of peroxide/kg fat.

### Thiobarbituric acid (TBA) reactive species assay

The products of lipid peroxidation were calculated by reaction of malondialdehyde (MDA) with TBA at 532 nm and the formation of MDA [23]. Soybean phosphatidylcholine liposomes (5 mg/mL in KH_2_PO_4_ - K_2_HPO_4_ a buffer) were sonicated under cooling conditions to yield a milky solution. The reaction mixture contained 500 μL of this mixture, 300 μL buffer, containing different concentrations of EO (Tween 80 as cosolvent for dissolving EO in buffer), 100 μLFeCl_3_ (1 mM), and 100 μL ascorbic acid (1 mM) to start peroxidation and was incubated for an hour at 37°C. Afterward, lipid peroxidation calculated by the reaction with TBA. TBA (1 mL, 1% in 50 mM NaOH) and acetic acid (1 mL, 20%) mixed and heated at 100°C for 30 min. In each tube, 5 mL butanol was added, vigorously vortexed and centrifuged at 1500 g for 15 min. The organic layer absorbance was measured at 532 nm. To calculate the level of inhibition of lipid peroxidation, the absorbance of the samples was compared with those of controls, which did not contain the oil using the following equation. All of the values were based on the percentage antioxidant index (AI %):


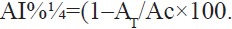


Where A_C_=The absorbance value of the fully oxidized control; A_T_=The absorbance of the test sample.

### Total volatile base nitrogen (TVBN) value

TVBN values were measured by the method of Association of Official Analytical Chemists [24]. Data are expressed in milligram of nitrogen per 100 g of sample.

### Statistical analysis

The data were analyzed using SPSS Version 18.0 (Windows; 10 SPSS Inc.), and all of the experiments were performed in triplicates. For the comparison of results among experimental groups analysis of variance (one-way ANOVA) was used. Tukey’s test was also used to compare the differences between mean values during the storage (p<0.05).

## Results

### Antioxidant activity of ZEOs and MEOs

ZEO had a higher phenolic content (263±4.35) than *M. piperita* (182±4.35). BCB, reducing, and chelating power of ZEO were 88.24±0.23, 19.42±0.13, and 88.13±4.08, respectively, which were higher than *M. piperita* (71.33±0.21, 16.71±0.12, and 69.48±3.90) EO. IC_50_ in DPPH assay was lower for *Z. multiflora* (6.28±0.23) than *M. piperita* (7.81±0.23) EO. Based on the above-mentioned values, *Z. multiflora* had higher antioxidant activity than MEO.

According to Table-1, BCB values of both EO were lower than BHA. Although, IC_50_ of reducing power of both of EOs was higher than BHA. ZEO (88.13±4.08) had a higher chelating power in comparison with quercetin (75.71±3.43), but *M. piperita* had a lower one (69.48±3.90). Table-1 shows the antioxidant potential of ZEOs and MEOs.

**Table-1:** Antioxidant potential of ZEO and MEOs using common antioxidant assays.

Antioxidant agent	DPPH IC_50_ (mg/ml)	Total phenolic contents	B carotene/linoleic acid bleaching assay	Reducing power IC_50_ mg/ml)	Chelating power at 0.5 mg/ml
*Z. multiflora*	6.28±0.23	263±4.35	88.24±0.23	19.42±0.13	88.13±4.08
*M. piperita*	7.81±0.23	182.00±4.35	71.33±0.21	16.71±0.12	69.48±3.90
BHA	0.68±0.23	----	89.28±0.23	2.13±0.11	----
Quercetin	----	----	----	----	75.71±3.43

BHA=Butylated hydroxyanisole, ZEOs and MEOs=*Zataria multiflora* and *Mentha piperita* essential oils

### Chemical analysis

pH values of the control and all the treatment groups did not show any significant change during the first 5 days of storage. In the control and all the treatment groups, pH values significantly increased at the 7^th^ and 9^th^ days of storage at 7°C (p<0.05) (Table-2).

**Table-2:** pH values of minced beef containing different levels of ZEO and MEOs for 9 days of storage at 7°C.

Day treatment	0	3	5	7	9
Control	5.6±0.13^Aa^	5.52±0.12^Aab^	5.31±0.12^Ab^	5.96±0.04^Ac^	6.39±0.09^Ad^
ZEO 0.3%	5.70±0.15^Aa^	5.70±0.11^Aa^	5.64±0.11^Aa^	6.12±0.14^Ab^	6.68±0.09^Bc^
ZEO 0.5%	5.63±0.07^Aa^	5.64±0.09^Aa^	5.61±0.12^Aa^	6.21±0.07^Ab^	6.74±0.17^Ac^
MEO 0.3%	5.53±0.26^Aa^	5.54±0.08^Aa^	5.48±0.13^Aa^	6.08±0.08^Ab^	6.71±0.09^Ac^
MEO 0.5%	5.58±0.1^Aa^	5.56±0.11^Aa^	5.52±0.13^Aa^	6.15±0.13^Ab^	6.82±0.09^Ac^

Same capital letters in each column means non-significant. Same small letter in each row means non-significant. ZEOs and MEOs=*Zataria multiflora* and *Mentha piperita* essential oils

**Figure-1:**
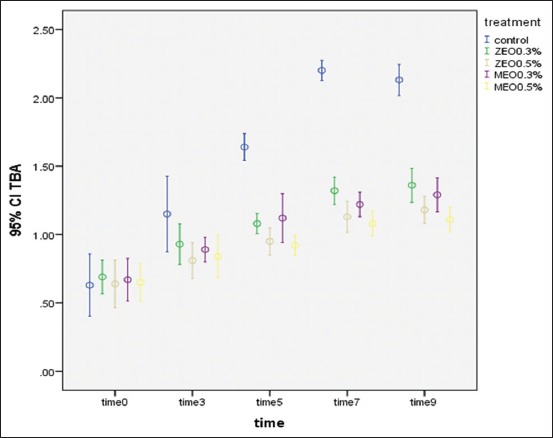
Thiobarbituric acid values of minced beef containing different levels of *Zataria multiflora* and *Mentha piperita* essential oils during 9 days of storage at 7°C.

TBA values of minced meat had a gradual ascending pattern during storage in the treatment groups, but in control one, they had a marked increase from the 5^th^ day of storage (p<0.05). There was a significant difference between the control and the treatment groups at the 5^th^, 7^th^, and 9^th^ days of storage at 7°C. TBA values significantly increased on the 5^th^ and 7^th^ days of storage in the control group (p<0.05). In other groups, TBA values did not have any significant change (Figure-1).

TVBN had a significant increase (p<0.05) in all groups. The least TVBN (25.14±0.28) belonged to ZEO 0.5% group on the 9^th^ day of storage at 7°C, but the control group had the highest one (34.79±0.35) (Table-3). PV value had a significant increase in all groups from the 3^rd^ day of storage. However, it had the least increase in ZEO 0.5% and MEO 0.5% treatment groups (Table-4).

**Table-3:** TVBN values of minced beef containing different levels of ZEOs and MEOs during 9 days of storage at 7°C.

Day treatment	0	3	5	7	9
Control	8.23±0.26^Aa^	16.29±0.31^Aa^	24.89±0.28^Ac^	29.84±0.30^Ad^	34.79±0.35^Ae^
ZEO 0.3%	7.94±0.31^Aa^	13.2±0.34^Bb^	18.43±0.27^Bc^	23.13±0.29^Bd^	27.63±0.27^Be^
ZEO 0.5%	8.12±0.32^Aa^	11.84±0.33^Cb^	15.13±0.26^Cc^	21.14±0.42^Cd^	25.14±0.27^Ce^
MEO 0.3%	8.24±0.33^Aa^	13.14±0.32^Bb^	17.94±0.28^Bc^	24.81±0.31^Dd^	28.23±0.27^Be^
MEO 0.5%	8.04±0.32^Aa^	11.64±0.29^Cb^	15.73±0.25^Cc^	20.84±0.31^Cd^	26.12±0.30^De^

Same capital letters in each column means non-significant. Same small letter in each row means non-significant. ZEOs and MEOs=*Zataria multiflora* and *Mentha piperita* essential oils, TVBN=Total volatile base nitrogen

**Table-4:** PVs of minced beef containing different levels of ZEOs and MEOs during 9 days of storage at 7°C.

Day treatment	0	3	5	7	9
Control	0.05±0.00*^a^	1.390±0.05^b^	1.66±0.03^c^	1.941±0.04^d^	2.09±0.05^Ae^
ZEO0.3%	0.02±0.00*^a^	1.04±0.04^Ab^	1.23±0.06^Ac^	1.53±0.4^Ad^	1.79±0.06^Be^
ZEO 0.5%	0.07±0.00*^a^	0.83±0.05^Bb^	1.02±0.06^Bc^	1.23±0.06^Bd^	1.54±0.04^Ce^
MEO0.3%	0.06±0.00*^a^	1.14±0.04^Ab^	1.19±0.08^Ab^	1.47±0.09^Ac^	1.65±0.03B^Cd^
MEO0.5%	0.10±0.00*^a^	0.74±0.08^Bb^	0.94±0.04^Bc^	1.28±0.08^Bd^	1.61±0.11^Ce^

Same capital letters in each column means non-significant. Same small letter in each row means non-significant.

* p-value was not stimulated because SDs are zero in each group. ZEOs and MEOs=*Zataria multiflora* and *Mentha piperita* essential oils, SDs=Standard deviations, PVs=Peroxide values

## Discussion

Use of natural antioxidant in food mixture is a new spectrum of research in food science. One of the main resources of these compounds is EO extracted from medicinal plants. Minced meat is a highly perishable/oxidizable matrix due to the large surface area, which could easily come in contact with oxygen [25]. Thus, the use of EOs with potential antioxidant activity may effectively reduce oxidative spoilage.

The antioxidant activity of medicinal plants is associated with the phenolic content of them. High redox potentials of phenolic compounds enable them to act as reducing agents, hydrogen donors, and singlet oxygen quenchers [1,26]. The phenolic content of ZEOs and MEOs used in this study was 263±4.35 and 182±4.35, respectively, which were higher than in some other studies [3,27]. There is a high association between the phenol content of plant extracts and antioxidant activity. In this regard, antioxidant activity and phenolic contents of the plant extracts had a linear correlation [1].

Regarding the complex reactive nature of phytochemicals, at least two methods must be used for determining the antioxidant activity of the plant EO [1]. Miguel [26] reported that antioxidants are known as a scavenger of free radicals to inhibit the lipid oxidation [26]. Therefore, the antioxidant activity of ZEOs and MEOs was determined by measuring the total phenolic content and three spectrophotometric methods including BCB, DPPH, and FRAP assays.

Several *in vitro* and *in vivo* studies have demonstrated the antioxidant capacity of these EOs [1]. Furthermore, Karabagias *et al*. [28] observed a reduction of meat oxidation by the use of thyme and oregano in fresh lamb meat and the shelf-life extension of this product [28].

Singh *et al*. [12] reported the antioxidant capacity of peppermint oil at 734 nm (%) as 89.4±6.3. DPPH free radical scavenging activity and reducing power (absorbance 700 nm) were 92.6±6.8 and 0.9±0.3 [12], respectively, which were lower than the results of the present study. Some authors reported the higher DPPH activity of peppermint oil, as Sharafi *et al*. [3] showed its highest 63.82±0.05% inhibition of DPPH activity with an IC_50_=3.9 µg/ml.

MEO revealed 93.9±1.68% inhibition of DPPH activity with an IC_50_=273 µg/ml [14]. De Sousa Barros *et al*. [13] observed that the MEOs had an IC_50_ of 5.72±0.06 mg/mL in the DPPH assay [13]. MEO had (p>0.05) the same inhibition level of lipid peroxidation with the synthetic antioxidant BHT and lower (p<0.001) than BHA [3]. A high correlation observed between DPPH scavenging activity and the total phenolic content of the *M. piperita* (r2>0.989) [29].

Dashipour *et al*. [30] investigated the antioxidant activity of carboxymethyl cellulose films containing ZEO. The control film showed negligible antioxidant activity, although the films showed a significant elevation of DPPH scavenging activity (p<0.05) following an increase in ZEO concentrations [30].

During the storage of meat, the pH value increased due to the breakdown of proteins. Use of EOs in the present study retarded the increment of pH which is in agreement with other studies [31,32]. Karabagias *et al*. [28] indicated that lamb meat containing thyme EO (0.1%) had a slightly lower pH on day 9 of storage that shows the partial protective action of thyme EO against meat decomposition [28].

The treated samples had significantly lower TBA values than the control (p<0.05) in a period of 9-day storage. TBA values of the control sample reached the final value of 2.32, whereas in the weakest treatment group with 0.1% ZEO reached to 1.36 mg MDA/kg sample. The results of the antioxidant activity in the present study were comparable with the results previously reported by Tajik *et al*. [9] who studied the antioxidant activity of *Z. multifora* EO in the buffalo patty. Furthermore, while the concentration of EOs was increased, TBA values had a lower ascending pattern, which was completely consistent with other former studies [9,15,30]. In a study, thyme EO inhibits the increase of TBA in lamb meat during storage [28].

Smaoui *et al*. [33] observed that the combination of MEO and BacTN635 led to a decrease in TBARS values in stored minced meat. Minced beef treated with EOs (*Mentha* and *Lavandula*) showed the lowest TBA values (lipid oxidation) in another study as well [34]. Honarvar *et al*. [31] showed that the TBA values of chicken meat were lowest in the treatment containing ZEO. In another word, the shelf life of chicken meat preserved by films containing ZEO improved from 3 to 9 days when compared to the control samples with no direct contact with the film [31], which is in agreement with the result of the present study.

In the present study, TVBN values increased during storage in all groups, and the highest and the lowest increase rates were observed in the control samples and the ZEO 0.5% treated samples, respectively. These results are in accordance with results of Choobkar *et al*. [32] who investigated the effect of ZEO and nisin on the quality control of the light salted fillets of silver carp.

PVs of the groups containing EOs had a lower increase rate in comparison with the control group. With the increase of ZEO concentration, PV had a less increase [32]. In another study, the same result was seen by the use of *Z. multiflora* on the shelf life of vacuum-packaged trout burgers [15].

## Conclusion

According to the reported results, lipid hydrolysis and oxidation occurred in the minced meat stored at 7°C during the period of study. Results of this study showed that treatments with the higher concentration of EOs had the greatest effect on preserving chemical quality and stability (pH, TBA, TVBN, and PV) of the fresh minced meat; although all of the treatments were significantly efficient in freshness stability of minced meat in comparison with the control group. Therefore, *Z. multifora* and MEOs had a good antioxidant activity and could retard the spoilage process.

## Authors’ Contributions

MR designed and performed the experiments; MH performed the experiments and analyzed the data. MA assisted in data analysis and drafting the manuscript; AA and BJ assisted in the design of the study and analyzed the data. TZ critically analyzed the data and drafting the manuscript. All authors read and approved the final manuscript.
